# Trends in HIV-1 pretreatment drug resistance and HIV-1 variant dynamics among antiretroviral therapy-naive Ethiopians from 2003 to 2018: a pooled sequence analysis

**DOI:** 10.1186/s12985-023-02205-w

**Published:** 2023-10-25

**Authors:** Mulugeta Kiros, Sirak Biset, Birhane Gebremariam, Gebrehiwet Tesfay Yalew, Woldaregay Erku Abegaz, Alene Geteneh

**Affiliations:** 1 Department of Medical Laboratory Science, CollegeofMedicineandHealth Sciences, Aksum University, Aksum, Ethiopia; 2 Department of Medical Microbiology, School of Biomedical and Laboratory Sciences, College of Medicine and Health Sciences, University of Gondar, Gondar, Ethiopia; 3 Department of Medical Laboratory Science, College of Medicine and Health Sciences, Adigrat University, Adigrat, Ethiopia; 4 Department of Microbiology, Parasitology, and Immunology, School of Medicine, Addis Ababa University, Addis Ababa, Ethiopia; 5 Department of Medical Laboratory Sciences, College of Health Sciences, Woldia University, Woldia, Ethiopia

**Keywords:** HIV, Genetic diversity, Drug resistance, ART-naive, Antiretroviral therapy, Pooled sequence analysis, Ethiopia

## Abstract

**Background:**

Ethiopia is among the highly HIV-affected countries, with reported 12,000 and 12,000 AIDS-related deaths and incidents as per reports from 2021. Although the country has made a promising progress in antiretroviral therapy, recent studies have indicated that pretreatment drug resistance (PDR) is alarmingly increasing, which has become a challenge for the effectiveness of HIV treatment. Epidemiologic data on PDR is necessary to help establish ART regimens with good efficacy. Thus, this systematic review aimed to determine the trend analysis of PDR among ART-naïve individuals along with HIV variant dynamics in Ethiopia.

**Method:**

HIV-1 pol sequences from studies conducted between 2003 and 2018 among ART-naïve Ethiopian individuals were retrieved from GenBank and analyzed for the presence of PDR mutations (PDRM) along with the analysis of HIV-1 variant dynamics. The Calibrated Population Resistance (CPR) tool Version 8.1 and the REGA HIV-1 Subtyping Tool Version 3 were used to determine the PDRM and HIV-1 genetic diversity, respectively.

**Result:**

We identified nine studies and analyzed 1070 retrieved HIV-1 pol sequences in this systematic review. The pooled prevalence of PDR was 4.8% (51/1070), including 1.4% (15/1070), 2.8% (30/1070), and 0.8% (9/1070) for nucleoside reverse transcriptase inhibitor (NRTI), non-NRTI (NNRTI), and protease inhibitor (PI) resistance, respectively. NRTI and NNRTI concurrent PDRM were observed among 0.2% (2/799) of the analyzed sequences. The overall PDR prevalence has been increasing over the years. Though the prevalence of the NNRTI, NRTI, and PI PDR also increased over the years, the NNRTI increment was more pronounced than the others, reaching 7.84% in 2018 from 2.19% in 2003. The majority (97%; 1038/1070) of the genetic diversity was HIV-1 subtype C virus, followed by subtype C’ (2%; 20/1038) and other subtypes (1%; 10/1038).

**Conclusions:**

According to this systematic review, the overall pooled prevalence of PDR is low. Despite the low prevalence, there has been an increasing trend of PDR over the years, which implies the need for routine surveillance of PDRMs along with preventive measures. Hence, this supports the recently endorsed transition of ART regimens from NNRTI to integrase strand transfer inhibitor-based regimens recommended by the WHO. In addition, this finding underscores the need for routine baseline genotypic drug resistance testing for all newly diagnosed HIV-infected patients before initiating treatment to halt the upward trend of PDR.

**Supplementary Information:**

The online version contains supplementary material available at 10.1186/s12985-023-02205-w.

## Introduction

Although decades have passed since the start of the pandemic, the Human Immunodeficiency Virus (HIV) remains a major public health threat globally. The COVID-19 pandemic exacerbated the challenge of HIV infection due to overstretched healthcare delivery, competition for resources (including financial and healthcare-providing professionals), and the synergistic effect of co-infection [1]. According to the UNAIDS 2022 report, approximately 38.4 million people were living with HIV (PLWH) worldwide during the year 2021. Among these, the eastern and southern African regions bear the highest burden of the illness, covering 54% (20.6 million) of the global estimate. Similarly, being situated in this region, Ethiopia is among the most highly affected countries, with 12,000 AIDS-related deaths and 12,000 new HIV infections in the same year [2].

Anti-retroviral therapy (ART) has been scaled up globally from 7.8 million in 2010 to 28.7 million people in December 2021, resulting in a reduction of the burden associated with the pandemic [2]. While this is an outstanding progress in terms of scale-up, the development of pretreatment HIV-1 drug resistance (PDR), which comes in parallel with ART, is becoming a challenge for ART effectiveness [3]. ART scale-up in Sub-Saharan Africa, where drug resistance testing is not routinely available, is also similarly associated with an increasing prevalence of PDR [4, 5].

In Ethiopia, the publicly funded ART program was started in 2005, and the number of people using ART has now scaled up from 58,405 in the beginning to around 436,000 in 2017 [6–9]. Despite this, however, recent reports show that the PDR is increasing [10]. Therefore, epidemiologic data on PDR is crucial to helping establish optimal empiric ART regimens, especially in countries like Ethiopia, where treatment options are limited and HIV drug resistance testing is absent. To the best of our knowledge, no review study shows the trend of PDR among ART-naive HIV-positive individuals in Ethiopia. Hence, this review has examined the trend of PDR among ART-naïve HIV-positive individuals along with the HIV-1 variant dynamics over the years in Ethiopia.

## Methods

### Sequence data source, selection criteria, and search strategy

**Fig. 1:**
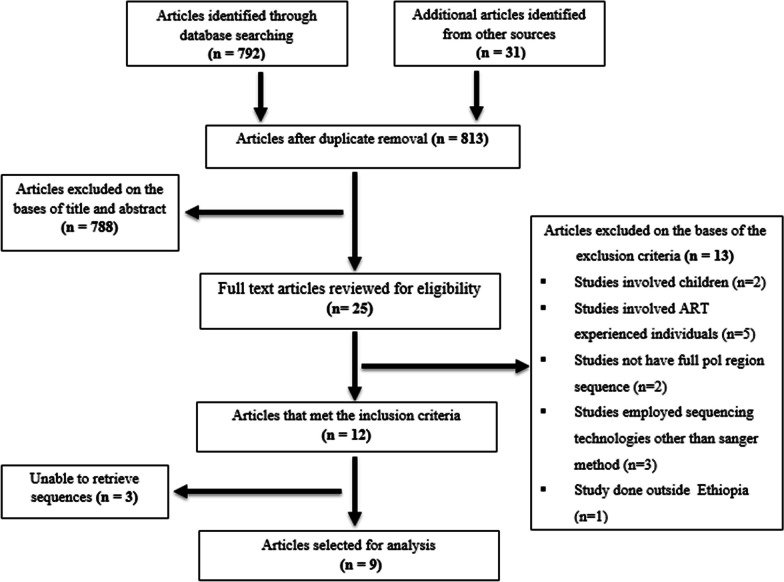
Flow diagram of studies identified and selected for the pooled sequence analysis

The online databases Google Scholar and PubMed were searched for PDR in Ethiopia to identify relevant literature published until March 30, 2023. The search for the literature was done using the following search terms: [(“human immunodeficiency virus” OR HIV OR AIDS OR “acquired immunodeficiency syndrome”) AND (“antiretroviral therapy” OR HAART OR ART) AND (“drug resistance” OR naïve OR "pregnant women" OR antenatal OR subtype OR surveillance) AND (Ethiopia)]. In addition, the International AIDS Society (IAS) Conference, and Conference on Retroviruses and Opportunistic Infections (CROI) databases were also searched for additional articles. Upon duplicate removal, all retrieved publications were reviewed to determine which studies contained data regarding PDR in Ethiopia. All PDR studies that involved ART-naïve HIV-infected individuals aged ≥ 14 years old in Ethiopia were included in this review. We excluded studies conducted in children, those that have incomplete sequences (did not contain complete PR (99 codons) and partial RT (the first 230 codons), and those studies that were done among ART-experienced individuals (Fig. 1).

### Sequence analysis

Sequences of the included studies were downloaded from GenBank if they were publicly available and, if they were not found online, study authors were contacted for the respective sequences. Where several sequences were found from the same research participant (for instance, in cohort studies), we only included the sequence from the earliest time point. Accordingly, we included 1070 sequences (from the total of 1226 retrieved sequences) that have full protease and partial RT. Before sequence analysis, quality control of the pooled sequences from these studies was done using the Calibrated Population Resistance (CPR) tool version 8.1, available in the Stanford HIVDB database (http://cpr.stanford.edu/cpr.cgi, (accessed on 02 April 2023)). Sequences (156 out of the total 1226 retrieved sequences) with poor quality based on the CPR quality tool were excluded from the analysis.

### Genotypic drug resistance, phylogenetic and statistical analysis

Genotypic PDR mutations (PDRMs) were determined using CPR tool version 8.1. (accessed on 05 April 2023) and were characterized according to the sampling year of the retrieved sequence data. We did not include sex and age information in the datasets or perform correlations between PDRMs and sociodemographic factors because most sequence annotations retrieved did not include this information. The HIV-1 subtype determination was done using the REGA HIV subtyping tool version 3.46 (Leuven University, Leuven, Belgium; https://www.genomedetective.com/app/typingtool/hiv, accessed on 04 April 2023). The sequence alignment and maximum likelihood phylogenetic tree construction was made using the Geneious Prime® 2023.0.4 Software (https://www.geneious.com) and was consecutively visualized by Interactive Tree Of Life (iTOL) version 6 (https://itol.embl.de/). PDR level classification (low: < 5%, moderate: 5–15%, or high: > 15%) was made based on the WHO threshold survey protocol [11].

## Results

We first identified 823 research articles and excluded 788 of the studies based on title and abstract review. The rest 25 full-text articles were further reviewed based on the eligibility criteria, which led to the selection of 12 articles for analysis, three of which were removed due to the unavailability of sequence data (Fig. 1).

### Prevalence and drug resistance mutation pattern of HIV

The pooled prevalence of PDR was 4.8% (51/1070). Thirty (2.8%; 30/1070) of the sequences harbored NNRTI PDRM, which is the highest proposition, followed by NRTI (1.4%; 15/1070) and PI PDRM (0.8%; 9/1070). NRTI and NNRTI concurrent PDRM were observed among two individuals (0.2%; 2/1070) of the analyzed sequences (Table 1).

**Table 1:** Proportion of sequences with PDRMs

Resistance category	No. analyzed	No. containing PDRM	%
Sequences with any PDRM	1070	51	4.8
PR sequences with any PI PDRM	1070	9	0.8
RT sequences with any NRTI PDRM	1070	15	1.4
RT sequences with any NNRTI PDRM	1070	30	2.8
RT sequences with any NRTI + any NNRTI PDRM	1070	2	0.2

*PDRM* pretreatment drug-resistance mutation, *PR* protease, *PI* protease inhibitor, *RT* reverse-transcriptase, *NRTI* nucleotide reverse-transcriptase inhibitor, *NNRTI* non-nucleotide reverse-transcriptase inhibitor

**Fig. 2:**
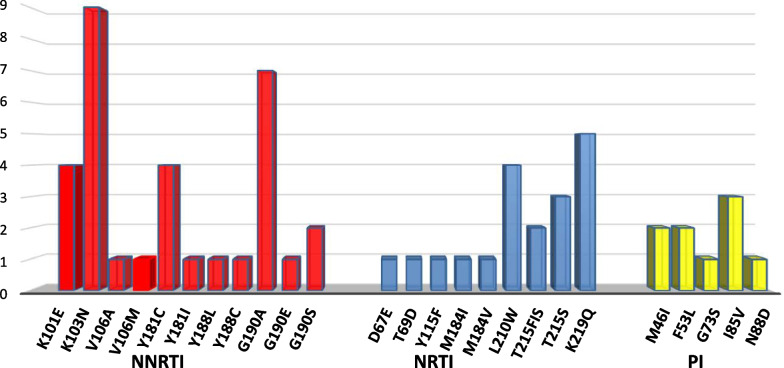
Prevalence of specific mutations in HIV-1 sequences with any drug resistance mutation. The horizontal axis includes all the mutations observed (NNRTI, NRTI, and PI-associated mutations in this review, while the vertical axis represents their respective frequency

The K103N NNRTI-associated mutation was the most prevalent observed in this review (17.6%; 9/51), followed by G190A (13.7%; 7/51), K101E (7.8%; 4/51), Y188C (7.8%; 4/51), and G190S (5.9%; 3/51). Concerning NRTI mutations, the most frequent mutations were: K219Q (9.8%; 5/51), L210W (7.8%; 4/51), T215S (5.9%; 3/51), and T215FIS (3.9%; 2/51). Among the PI-associated major mutations, the I85V mutation was the most prevalent mutation observed in this review (5.9%; 3/51), followed by M46I and F53L, which were detected in two individuals each (3.9%; 2/51) (Fig. 2). Among the individuals who harbored any PDRMs, four harbored more than one NNRTI-associated mutation, while two harbored more than one NRTI-related mutation simultaneously. The remaining 45 individuals only harbored a single PDRM at a time. For an extensive review of all identified PDRMs, see Additional file 1: Table S1.

### Magnitude and trend of PDR over time

**Fig. 3:**
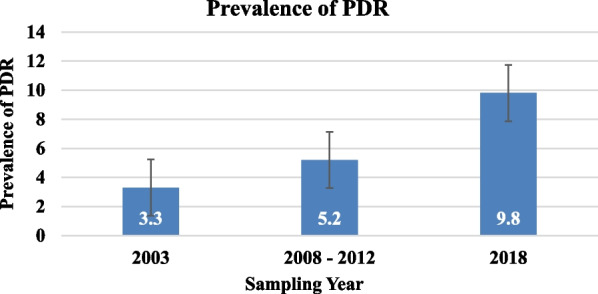
Prevalence of PDR over time

**Fig. 4:**
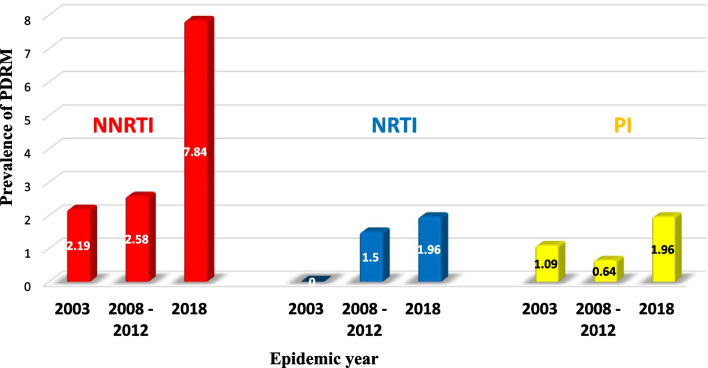
Prevalence of NNRTI, NRTI, and PI PDRM over time

To assess the trend of PDR over time, we pooled sequences based on the sampling year. Accordingly, we were able to observe an increment in PDR prevalence from low in 2003 (3.3%) to moderate (5.2%) in 2008–2012. This increase continued and reached 9.8% in 2018, which shows an overall increment in PDR prevalence from 2003 to 2018 (Fig. 3). The NNRTI PDRM was the most prevalent mutation observed over the years relative to the NRTI and PI PDRMs. The trend of NNRTI, NRTI, and PI PDRMs has increased over the years, reaching a peak prevalence in 2018; 7.8%, 1.96%, and 1.96%, respectively (Fig. 4).

### HIV variant dynamics and transmission cluster characteristics

**Fig. 5:**
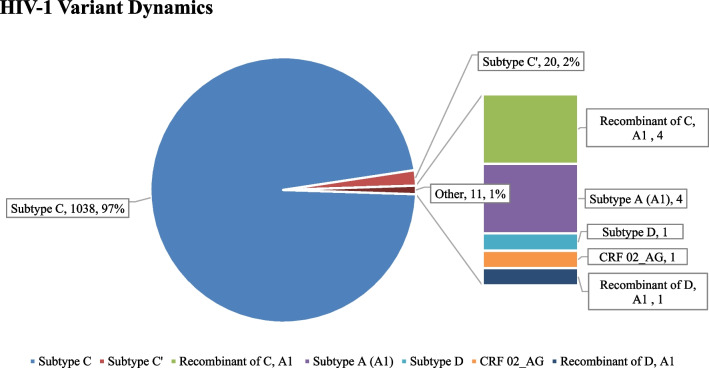
HIV-1 variant dynamics in Ethiopia

The REGA HIV subtyping tool indicated that most of the sequences analyzed (97%; 1038/1070) were HIV-1 subtype C virus followed by subtype C’ (2%; 20/1070), and others (1%; 10/1070) (see Fig. 5). Regarding the geographical distribution of the variants in all study areas across the nation, the HIV-1 subtype C virus remains the major clade, accounting for more than 97% of the total sequences. The majority (80%) of the HIV-1 subtype C-like clade was mainly observed in the northwest of the country, while the other subtypes were dispersed across the nation.

**Fig. 6:**
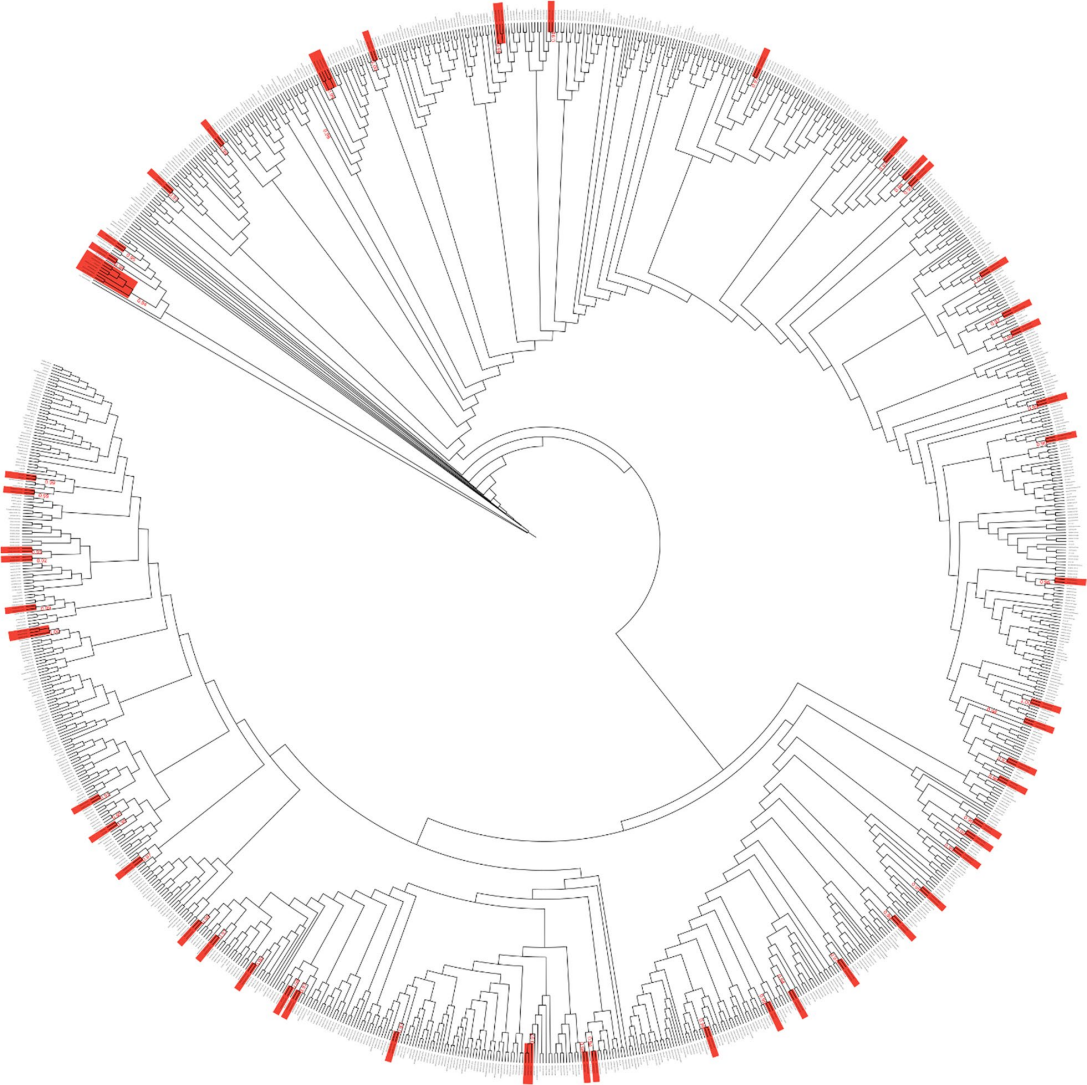
Maximum likelihood phylogenetic tree depicting the transmission clusters. The ML tree was constructed using Geneious Prime® 2023.0.4 with the Kimura 2 parameter. Bootstrapping was performed with 1000 replicates. Transmission clusters with a bootstrap value of 90% and above are seen shaded in red in the tree

To depict transmission clusters, the 1070 sequences, along with other additional reference sequences (A-K and CRFs; Table S2 in Additional file 2) from the Los Alamos HIV sequence database (https://www.hiv.lanl.gov/) were aligned and a maximum likelihood (ML) phylogenetic tree was constructed using Geneious Prime® 2023.0.4 Software (https://www.geneious.com). The neighbor-joining method with 1000 bootstrap replicates under Kimura’s 2-parameter correction was employed. Accordingly, we identified 50 transmission clusters in total (Fig. 6). Those transmission clusters that have a bootstrap value ≥ 90% only are shown in the tree below.

## Discussion

Currently, PDR has become a challenge to the success of ART, which makes HIV prevention very difficult. According to the WHO's recent report, PDR prevalence is increasing globally [12]. The same is true for low and middle-income countries, which have shown a substantial increment recently [4]. Therefore, determining PDR at the population level is crucial for optimizing patient and population-level treatment outcomes that will consequently help achieve the third WHO/UNAIDS 90–90–90 target. This review provided the first nationally representative description of the PDR trend along with the HIV variant dynamics over the years.

The pooled prevalence of PDR among ART-naïve individuals in Ethiopia in this systematic review was 4.8%, which is low as per the WHO HIV drug resistance classification [11]. This finding is in line with a similar study from China [13, 14], reports from Tanzania [15], and Kenya [16]. It is, however, lower than the rate of PDR reported from South Africa [17], Cameroon [18], Namibia [19], Brazil [20], Italy [21], and England [22].

The NNRTI-associated PDRMs were the most frequently observed mutations in this review, which corroborates observations from different studies across the globe [23–28]. This finding supports the recent transition of the ART regimen in Ethiopia from NNRTI to WHO-recommended integrase strand transfer inhibitor-based regimens. The K103N mutation, which is a non-polymorphic mutation that confers a high-level resistance against efavirenz (EFV) and nevirapine (NVP) [29, 30], was found in a high proportion (17.6%; 9/51) from the NNRTI associated PDRMs. This is similar to reports from Malawi [26], Israel [23], Brazil [20], Tanzania [15], Namibia [19], and Italy [21]. The G190A (13.7%; 7/51) mutation, which is equally selected in persons receiving NVP and EFV [31, 32] was also a common NNRTI-associated mutation, followed by the K101E mutation (7.8%; 4/51), Y188C (7.8%; 4/51) and G190S (5.9%; 3/51) (Fig. 2). The K101E and Y188C mutations are associated with reduced susceptibility to NVP, Etravirine (ETR), and Rilpivirine (RPV) [33–35], while Y188C and G190S reduce susceptibility to NVP and EFV [33, 35–37].

Among the NRTI-associated mutations, K219Q and L210W mutations which are selected by the thymidine analogs zidovudine (AZT) and Stavudine (d4T), were reported in high frequencies (9.8% (5/51) and 7.8% (4/51)), respectively. In addition to these mutations, others like T215S (5.9%; 3/51) and T215FIS (3.9%; 2/51) were also observed in this review, although in relatively lower frequencies. T215S/I mutation is associated with an increased risk of virologic failure associated with AZT or d4T usage, while T215F confers reduced susceptibility to all currently approved NRTIs except emtricitabine (FTC) and lamivudine (3TC) [38]. It is known that AZT and d4T are commonly used in the country similar to other resource-limited settings; hence, the presence of the mentioned mutations underscores the need for routine genotypic resistance testing.

Protease inhibitor (PI) mutations are usually observed at lower frequencies in different studies [23–28, 39]. Likewise, the frequency of PI mutations is low (0.8%) in this review. The mutations observed are I85V (5.9%; 3/51), which was the most prevalent PI mutation, followed by M46I (3.9%; 2/51) and F53L (3.9%; 2/51). The I85V is a non-polymorphic mutation selected by Nelfinavir (NFV) and Atazanavir (ATV) [40, 41] while the M46I and F53L are associated with reduced susceptibility to ATV and Lopinavir (LPV) [42, 43]. M46I, a non-active site mutation in HIV-1 protease, has been clinically associated with saquinavir (SQV) resistance in HIV patients [44]. Altogether, the mentioned mutation profile in this review indicates that regimens containing NFV, ATV, and LPV are more likely to be effective relative to their NNRTI and/or NRTI counterparts.

Concerning the PDR prevalence per year, the overall PDR prevalence was observed to increase from 3.3% in 2003 to 9.8% in 2018 (Fig. 3). The accumulation of the PDRMs over time might have led to the upward trend of PDR over the years. In line with this finding, a similar increment has also been reported in other studies from Mozambique [45] and South Africa [17]. In addition, the NNRTI-associated PDRMs also increased over time (from 2.19% in 2003 to 7.84% in 2018) (Fig. 4), which is once again similar to observations from other studies around the world [23–28, 39].

The current study confirmed the HIV-1 subtype C virus as the predominant clade in Ethiopia, similar to other eastern and southern African countries like South Africa [46], Mozambique [45], Malawi [26], and Botswana [47]. This class accounts for 97% of the pandemic in the country. Despite being low, the influx of other variants, including HIV-1 subtype C-like (2%, 20/1070), recombinant of C and A1 (0.38%, 4/1070), HIV-1 subtype A (A1) (0.38%, 4/1070), HIV-1 subtype D (0.09%, 1/1070), HIV-1 CRF 02_AG (0.09%, 1/1070), and recombinant of D and A1 (0.09%, 1/1070), has been observed (Fig. 5). The occurrence of these other variants indicates the possible introduction of other HIV-1 subtypes from neighboring countries. Therefore, preventive measures should always be there to prevent the possibility of further introduction of new subtypes. The 50 transmission clusters found in the sequences analyzed suggest that many HIV-positive individuals engage in risky sexual behavior, emphasizing the necessity of a thorough understanding of the transmission dynamics of HIV-1 subtype C in the population. An increased public health intervention program aimed at these people is also necessary to stop such potentially risky behaviors that will negatively affect HIV transmission. The current review has some limitations. Firstly, all data used in the analysis were retrieved from the literature, and hence we were unable to analyze the correlation of drug resistance with demographic and clinical characteristics, as this information was not available for the majority of sequences. Secondly, in the current review, the studies were mainly from the capital city and from the northern part of the country, and hence some regions might have been underrepresented (due to absence of study), which might lower the representativeness of the study.

## Conclusions

In this systematic review, we presented temporal trends of HIV-1 PDR among ART-naïve individuals along with HIV-1 variant dynamics in Ethiopia. Despite the decade-long pandemic, the HIV variant dynamics remained the same, with HIV-1 subtype C still accounting for the majority of the clade (> 97%). Our analysis showed that the overall pooled prevalence of PDR is borderline low (4.8%) 15 years after the rollout of ART in Ethiopia. However, the trend has been seen to be increasing over the years, which is concerning and indicates the need for routine surveillance of PDRMs along with consecutive preventive measures in the country. In addition, the observed increasing trend in NNRTI PDR supports the recent transition of the ART regimen from NNRTI to WHO-recommended integrase strand transfer inhibitor-based regimens.

### Supplementary Information

**Additional file 1. Table S1**: List of PDRMs identified in the current study.

**Additional file 2. Table S2**: List of Reference sequences used in the current study.

## Data Availability

All data generated or analyzed are included in this manuscript and its supplementary information files.

## Abbreviations

AIDS: Acquired immunodeficiency syndrome
ART: Antiretroviral treatment
ATV: Atazanavir
AZT: Zidovudine
D4T: Stavudine
EFV: Efavirenz
ETR: Etravirine
FTC: Emtricitabine
HIV: Human immunodeficiency virus
LPV: Lopinavir
NFV: Nelfinavir
NNRTI: Non-nucleoside reverse-transcriptase inhibitor
NRTI: Nucleoside reverse-transcriptase inhibitor
NVP: Nevirapine
PDR: Pretreatment drug resistance
PDRM: Pretreatment drug resistance mutation
PI: Protease inhibitor
PLWH: People living with HIV
PR: Protease
RPV: Rilpivirine
RT: Reverse-transcriptase
SQV: Saquinavir
3TC: Lamivudine
WHO: World health organization
