# Supplementary material for: Trends in HIV-1 pretreatment drug resistance and HIV-1 variant dynamics among antiretroviral therapy-naive Ethiopians from 2003 to 2018: a pooled sequence analysis
Source: Virol J. 2023 Oct 25;20:243. doi: 10.1186/s12985-023-02205-w (PMC10601210; doi:10.1186/s12985-023-02205-w)
Supplement: Supplementary file 1 — Additional file 1. Table S1: List of PDRMs identified in the current study. [file 12985_2023_2205_MOESM1_ESM.docx]

**Supplementary table S1**

**List of PDRMs identified in the current study**

| **Sequence Header** | **NRTI PDRMs** | **NNRTI PDRMs** | **PI PDRMs** |
| --- | --- | --- | --- |
| AB285807.1: ETH-G-135 | - | G190A |  |
| AB285798.1: ETH-G-109 | - | G190A | - |
| AB285793.1: ETH-G-094 | - | - | I85V |
| KU319549.1 ET165 | - | - | I85V |
| KU319529.1 | T215S, K219Q | - | - |
| KF026184.1 ETH-G-5763 | - | - | F53L |
| KF026168.1 ETH-G-5712 | - | G190A | - |
| KF026166.1 ETH-G-5710 | L210W | - | M46I |
| KF026135.1 ETH-G-5604 | D67E | - | - |
| KF026098.1 ETH-G-5533 | - | G190A | - |
| KF026073.1 ETH-G-5496 | L210W | - | - |
| KJ807721.1 M075 | - | - | I85V |
| KJ807694.1 G025 | M184I | Y181I | - |
| KJ807688.1 M086 | - | G190A | - |
| KJ807667.1 G174 | - | G190E | - |
| KJ807657.1 G043 | - | - | M46I |
| KJ807653.1 G014 | - | G190A | - |
| MG010044.1 ETH144 | T215S | - | - |
| MG010031.1 ETH646 | - | Y181C, Y188C | - |
| MG009972.1 ETH218 | K219Q | - | - |
| MG009962.1 ETH042 | - | Y181C | - |
| MG009939.1 ETH484 | - | K103N | - |
| MG009929.1 ETH038 | T215S | - | - |
| MG009904.1 ETH479 | K219Q | - | - |
| MG009889.1 ETH205 | T215FIS, K219Q | - | - |
| MG009857.1 ETH354 | T69D | - | - |
| MG009820.1 ETH027 | - | Y181C | - |
| MG009810.1 ETH465 | T215FIS | - | - |
| MG009808.1 ETH406 | - | K103N | - |
| MG009784.1 ETH318 | - | - | F53L |
| MG009744.1 ETH368 | - | G190A | - |
| MG009719.1 ETH020 | L210W | - | - |
| MG009697.1 ETH196 | - | - | N88D |
| MG009631.1 ETH216 | L210W, K219Q | - | - |
| MG009611.1 ETH604 | - | G190S | - |
| KJ561135.1 HIV-1 isolate 9_Baseline | - | K103N | - |
| KJ561131.1 HIV-1 isolate 346_ Baseline | - | K101E | - |
| KJ561128.1 HIV-1 isolate 300_ Baseline | - | K103N, V106M | - |
| KJ561125.1 HIV-1 isolate 243_ Baseline | - | K103N | - |
| OL598850.1 ADR-008 | - | K101E | - |
| OL598755.1 ADR-078 | - | K101E | - |
| OL598738.1 ADR-099 | - | K103N | - |
| MH324999.1 ETHTDRGON088 | - | Y181C | - |
| MH324967.1 ETHTDRGON058 | - | K103N | - |
| MH324965.1 ETHTDRGON056 | - | G190S | - |
| MH324964.1 ETHTDRGON055 | - | K103N | - |
| MT416698.1 NI-033A | - | K103N | - |
| MT416681.1 AL-084 | - | K101E | - |
| MT416676.1 AL-078 | - | - | G73S |
| MT416672.1 AL-074 | Y115F, M184V | Y188L | - |
| MT416667.1 AK-135 | - | V106A | - |
