# Supplementary material for: Trends in HIV-1 pretreatment drug resistance and HIV-1 variant dynamics among antiretroviral therapy-naive Ethiopians from 2003 to 2018: a pooled sequence analysis
Source: Virol J. 2023 Oct 25;20:243. doi: 10.1186/s12985-023-02205-w (PMC10601210; doi:10.1186/s12985-023-02205-w)
Supplement: Supplementary file 2 — Additional file 2. Table S2: List of Reference sequences used in the current study. [file 12985_2023_2205_MOESM2_ESM.docx]

**Supplementary table S2**

**List of Reference sequences used** **in the current study**

| **Genbank Accession Number** | **Clade** | **Country^1^** | **Year^2^** |
| --- | --- | --- | --- |
| AB032740 | 01_AE | TH | 1995 |
| AF286226 | 07_BC | CN | 1997 |
| MH909568 | 100_01C | CN | 2013 |
| MH705151 | A | CD | 1987 |
| HXB2-K03455 | B | FR | 1983 |
| AY727522 | C | BR | 2004 |
| AF110960 | C | BW | 1996 |
| AY713417 | C | ET | 2002 |
| AB023804 | C | IN | 1993 |
| AF286234 | C | TZ | 1998 |
| AF1338211MB2059 | D | KE | 1993 |
| AF0757031FIN9363 (F1) | F | FI | 1993 |
| AY371158102CM0016BBY | F2 | CM | 2002 |
| MH705162 | G | CD | 1987 |
| AF190127 | H | BE | 1993 |
| AF082394 | J | SE | 1993 |
| AJ249239 | K | CM | 1996 |

^1^Country: two-letter country code (iso 3166-2); <https://datahub.io/core/country-list>

^2^Year: Year of sample collection
